# Mapping definitions of co‐production and co‐design in health and social care: A systematic scoping review providing lessons for the future

**DOI:** 10.1111/hex.13470

**Published:** 2022-03-23

**Authors:** Daniel Masterson, Kristina Areskoug Josefsson, Glenn Robert, Elisabeth Nylander, Sofia Kjellström

**Affiliations:** ^1^ Jönköping Academy for Improvement of Health and Welfare, School of Health and Welfare Jönköping University Jönköping Sweden; ^2^ Department of Behavioural Science Oslo Metropolitan University Oslo Norway; ^3^ Faculty of Health Studies VID Specialized University Oslo Norway; ^4^ Florence Nightingale Faculty of Nursing, Midwifery & Palliative Care King's College London London UK; ^5^ Jönköping University Library Jönköping University Jönköping Sweden

**Keywords:** co‐design, co‐production, definitions, healthcare, principles, social care, systematic scoping review, values

## Abstract

**Objectives:**

This study aimed to explore how the concepts of co‐production and co‐design have been defined and applied in the context of health and social care and to identify the temporal adoption of the terms.

**Methods:**

A systematic scoping review of CINAHL with Full Text, Cochrane Central Register of Controlled Trials, MEDLINE, PsycINFO, PubMed and Scopus was conducted to identify studies exploring co‐production or co‐design in health and social care. Data regarding date and conceptual definitions were extracted. From the 2933 studies retrieved, 979 articles were included in this review.

**Results:**

A network map of the sixty most common definitions and—through exploration of citations—eight definition clusters and a visual representation of how they interconnect and have informed each other over time are presented. Additional findings were as follows: (i) an increase in research exploring co‐production and co‐design in health and social care contexts; (ii) an increase in the number of new definitions during the last decade, despite just over a third of included articles providing no definition or explanation for their chosen concept; and (iii) an increase in the number of publications using the terms co‐production or co‐design while not involving citizens/patients/service users.

**Conclusions:**

Co‐production and co‐design are conceptualized in a wide range of ways. Rather than seeking universal definitions of these terms, future applied research should focus on articulating the underlying principles and values that need to be translated and explored in practice.

**Patient and Public Contribution:**

The search strategy and pilot results were presented at a workshop in May 2019 with patient and public contributors and researchers. Discussion here informed our next steps. During the analysis phase of the review, informal discussions were held once a month with a patient who has experience in patient and public involvement. As this involvement was conducted towards the end of the review, we agreed together that inclusion as an author would risk being tokenistic. Instead, acknowledgements were preferred. The next phase involves working as equal contributors to explore the values and principles of co‐production reported within the most common definitions.

## 1 INTRODUCTION

During the last decade, there has been an exponential growth of academic publications on all aspects of co‐production^1^ and an increase in wider discourses in the use of the term ‘co‐production’ itself.^2^ Co‐production has been described as a broad umbrella term^3^ and as a term to cover a range of ‘co’‐words^4^, ^5^ that are often ambiguous and appear to be applied to a wide range of contexts, activities and actors.^6^ Consequently, co‐production has been considered a slippery,^7^ woolly^8^ and muddled^6^ concept, the benefits of which may be diminished if the definition is unclear or misapplied. Alongside this increasing interest in co‐production, there has been a phenomenon for which Williams et al.^9^ use the term ‘cobiquity’ to describe ‘an emergence of a plethora of “co”‐words, promoting a conflation of meanings and practices from different collaborative traditions’ (p 2). For example, the term co‐design has been considered interchangeable with co‐creation,^10^ co‐production^11^ and described as a co‐form within the co‐production umbrella^5^ as well as been described as a specific instance of co‐creation.^12^ While the term ‘co‐production’ is largely credited to Ostrom et al.,^13^ co‐design has its own distinct disciplinary origins and features that can be traced back to the participatory design movement in Scandinavia in the 1970s.^14^ Here, we seek to acknowledge both the similarities and differences between co‐production and co‐design while exploring their—often closely related—use in health and social care contexts.

At first sight, it may seem that adequate consideration has been given to such issues; yet, there remains a lack of clarity on the meaning of co‐production and co‐design in the specific context of health and social care. To illustrate this, published reviews refer to over 30 separate definitions for co‐production and related concepts in relation to healthcare,^15^ acute healthcare,^16^ public care services,^17^ management,^18^ information and communication technologies^19^, ^20^ as well as proposed benefits of co‐production such as communication^21^ and cost‐efficiency.^22^ There have also been reviews exploring co‐production within specific populations as well as associated concepts such as community engagement,^23^ shared decision‐making,^24^ parental engagement,^25^ user involvement,^26^ patient involvement,^27^ patient and public involvement,^28^ patient engagement,^29^ patient advisors and patient engagement^30^ and patients as coresearchers.^31^ As noted by Locock and Boaz,^32^ there is a ‘crowded landscape’ of definitions and various approaches alongside an ongoing debate as to what counts as meaningful involvement. The description of the participants who engage in co‐production and co‐design varies depending on the adopted definition for these concepts and the context in which they are applied. The extent of this engagement and who is considered to be a relevant stakeholder can also vary. Our systematic scoping review focuses specifically on the relationship between the terms ‘co‐production’ and ‘co‐design’ as applied in the health and social care contexts that seeks to encapsulate previous work. Noting the systematic reviews undertaken on the concepts of co‐production and co‐creation,^10^, ^17^ we set out to systematically map this complex landscape by focusing on how the concepts of co‐production and co‐design relate to each other and how they have evolved over time. Further, our review will seek to identify the proportion of studies involving service users and that which does not.^9^

### 1.1 Objectives

The aim of this systematic scoping review was to explore co‐production and co‐design terminology in the context of health and social care by (1) exploring the use of the terms co‐production and co‐design over time and (2) providing a comprehensive network map of the most common definitions of co‐production and co‐design.

## 2 METHODS

As described in the Samskapa research programme protocol,^33^ a systematic scoping review was undertaken to establish ‘what is out there’ in relation to co‐production and co‐design within health and social care services. A scoping review is a preliminary assessment of the literature for potential size and scope with the aims of identifying the nature and extent of research evidence.^34^ Following the Johanna Briggs Institute methodology for scoping reviews^35^ and the PRISMA‐ScR checklist^36^ (File S1), we explore the definitions of the two concepts under study (co‐production and co‐design), the contexts in which they have been applied (within health and social care) and the participants involved.

### 2.1 Concept and context

We began by exploring key terms used in seminal articles identified through consultation with leading experts in the fields of co‐production and co‐design. We also collated key terms from research and search terms used in previous reviews (File S2). We conducted a series of pilot searches in Scopus and PubMed that were guided by a research librarian with expertize in searching academic databases. The following terms were identified as the most efficient search terms and most relevant to our review aims.

co‐produc*ORcoproduc*ORco‐design*OR codesign*

The use of truncation (*) was used to ensure that articles with terms such as ‘co‐designers’ and ‘co‐producers’ were also included. We acknowledge that there are a range of other word variations of ‘co’ that we also considered in our pilot searches (Files S2 and S3); however, in keeping with our research aims, we focused on locating studies using the term co‐production and/or co‐design in their title and/or abstract and/or key words. The context of this systematic scoping review is health and social care services. Our focus on health and social care services is addressed by incorporating these terms into the search strategy (health OR social OR ‘public service*’ OR ‘public sector’) as well as our exclusion criteria (File S4).

### 2.2 Information sources and search strategy

The following databases/platforms were searched by a research librarian: CINAHL with Full Text (EBSCOHost), Cochrane Central Register of Controlled Trials (Wiley), MEDLINE (EBSCOhost), PsycINFO (ProQuest), PubMed (legacy) and Scopus (Elsevier). Our full search strategy is reported in File S4.

#### 2.2.1 Eligibility criteria

We adopted an inclusive approach regarding primary and secondary research, including all methodologies, conceptual papers and articles exploring co‐production or co‐design. There were no limitations concerning dates.

**Figure 1:**
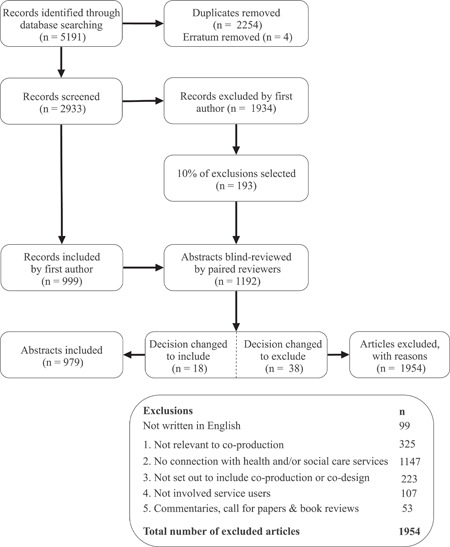
PRISMA flow diagram. A flow diagram showing the selection of evidence and reasons for exclusion. PRISMA, Preferred Reporting Items for Systematic Reviews and Meta‐Analyses

The establishment of the inclusion and exclusion criteria (Figure 1) was an iterative process shaped by a series of pilot searches and discussions to reduce the number of irrelevant articles. Given the practical challenges of language translation and our focus on the terms co‐production and co‐design, articles were required to be written in the English language. Our first inclusion criterion was designed to ensure that the use of the term ‘co‐production’ or ‘co‐design’ was relevant to our concept of interest (i.e., not in relation to genetics and cell structure). Our second criterion was to ensure that articles were relevant to our focal context of health and social care services. From our pilot search, we established that our search terms were locating articles recommending co‐production in their conclusion without having studied or discussed it in any depth before this recommendation, so we added the third criterion to remove these. Our fourth criterion sought to ensure that the included articles involved service users (rather than services being co‐produced solely with other professionals or organizations). For the purposes of this review, we refer to ‘service user’ as individuals engaged in any collaborative process that extends beyond the usual, direct provider–client relationship and may consist of individuals or groups of people who identify as citizens, patients, family member or carers. This term ‘service user’ was chosen following consultation with patient and public contributors. We considered service providers to be anyone whose professional role and responsibilities broadly represent the delivery of health and social care services. These could include health and social care professionals, administrative roles, support roles, teaching, training, research or any person who has formalized authority or can influence the process or quality of care given to a service user directly or indirectly. The fifth criterion was to exclude commentaries, editorials, call for papers and book reviews that did not progress or expand a conceptual definition. This was decided by reading the full text of these articles, and when a contribution to conceptual development was presented, these were included as conceptual papers.

### 2.3 Selection of sources of evidence

The selection process for this systematic scoping review is presented in a PRISMA flow chart (Figure 1). The results of the platform searches were deduplicated using EndNote (E. N.). The title and abstracts of records were then reviewed in Rayyan (D. M.). When a decision could not be made based on the abstract, the full article was reviewed. The inclusion and exclusion decisions were then reviewed by the wider team to ensure reliability of decisions. To achieve this, we randomly selected 10% of all inclusion/exclusion decisions and imported these into a new Rayyan review. Randomization was accomplished using Microsoft Excel to assign a random number to each article and sorting them until the 10% quota was met. This selection of articles was then randomly allocated to four reviewer pairs (researchers from the Samskapa programme) using the same method. All reviewers (see acknowledgements) had experience within the field of co‐production or co‐design and either had previous experience or received training in undertaking systematic reviews. This identified an agreement rate of 86% with the first author, but as this process identified several decision changes, all remaining included articles were reviewed by the wider team, after which we met to discuss conflicts. Each team documented conflicts in Rayyan and these were discussed to clarify the views from each team, before the first author made the final decision based on the discussions. Discussions revealed that the majority of conflicts were due to missing or overlooked information, and consensus was often reached through the discussion. Twelve conflicts were identified between the wider group, which were related to criterion two (context), where the first author made the final decision to reject 1 article and the remaining 11 were included.

### 2.4 Data charting process

Study characteristics and definitions were extracted by reading the abstract or full text. As ‘co‐words’ were occasionally used interchangeably, articles were allocated to either co‐production or co‐design based on the most common word variations of ‘co‐’ documented from the title, abstract and key words. Definitions of co‐production and co‐design were manually and purposefully extracted by reading the full articles included in this review and extracting the citation(s) used to define the adopted concept(s) (i.e., citation(s) alongside keyword(s), description(s) or definition(s) or part of a series of definitions). If provided, definitions by the author of each included article were also extracted. Extracted citations were screened to check for referencing errors and duplicates. Consistent with the procedure for scoping reviews, we did not assess the methodological quality of individual articles.^36^ We also did not judge the quality and depth of the co‐production or co‐design process beyond the stipulation that service users had been directly involved.

### 2.5 Synthesis of evidence

Data synthesis consisted of two approaches. A tabular record of included studies and associated definitions provided an opportunity to convey the scope of research in this field. Second, we undertook a data‐driven process to identify the most common definitions in the included articles and to explore how they relate to each other. The first author led the synthesis work and collaborated in analysing the findings with the whole team, in the steps of the process described below.

## 3 RESULTS

### 3.1 Characteristics and sources of evidence

**Figure 2:**
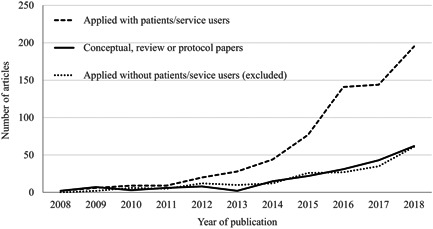
Applied and conceptual research during the last decade. A line chart showing the included articles exploring co‐production and co‐design in health and social care published in peer‐review journals between 2008 and 2018. These have been separated into applied research with services users (dashed lines), conceptual papers, reviews and protocols (solid line) and applied research with service users (dotted line)

From the 979 articles retrieved and included in this review, the majority (*n* = 580) referred primarily to co‐production and the remaining referred primarily to the term co‐design (*n* = 399). The included articles were published in over 400 journals with the top 20 journals presented in Table 1. We found that there has been an increase in applied research (*n* = 747), conceptual papers (*n* = 129), protocols (*n* = 65) and reviews (*n* = 38) exploring co‐production and co‐design in health and social care published in peer‐review journals, with an exponential increase between 2008 and 2018. A key finding based upon the application of one of our exclusion criteria (not involving citizens/service users) was that there has been an increase in the number of publications (*n* = 107) using the terms co‐production or co‐design while not involving citizens/patients/service users (Figure 2).

**Table 1:** Sources for included articles

Sources for included articles (top 20)	
	Co‐production	Co‐design	Combined
*International Journal of Integrated Care (IJIC)*	21	28	49
*BMJ Open*	17	19	36
*Health Expectations*	7	13	20
*Public Management Review*	19	1	20
*BMC Health Services Research*	6	13	19
*Mental Health & Social Inclusion*	17	2	19
*JMIR Research Protocols*	5	13	18
*Studies in Health Technology & Informatics*	7	11	18
*Implementation Science*	7	4	11
*Research Involvement and Engagement*	10	1	11
*Journal of Health Organization & Management*	10	0	10
*Journal of Mental Health Training, Education & Practice*	7	3	10
*Journal of Psychiatric & Mental Health Nursing*	9	1	10
*JMIR Mental Health*	2	7	9
*Mental Health Review Journal*	8	1	9
*Dementia*	4	4	8
*Health & Social Care in the Community*	6	2	8
*Health Research Policy & Systems*	7	1	8
*Journal of Integrated Care*	7	1	8
*Working with Older People Community Care Policy & Practice*	4	4	8

### 3.2 Definitions of co‐production and co‐design

**Figure 3:**
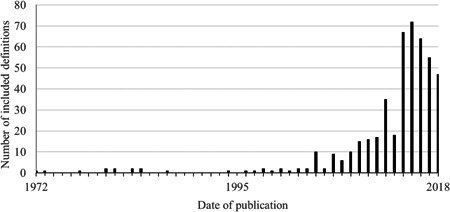
Publication date for included definitions. Dates of publication for each included definitions were plotted on a line chart to visualize when these definitions were first published

Of these included articles, 627 provided at least one definition when discussing co‐production (*n* = 425) or co‐design (*n* = 202). It is noteworthy that one‐third (*n* = 352) of the included articles provided no definition or explanation and relied on the reader to understand the meaning of either or both terms. Of those that had no definition, the majority used the term co‐design (*n* = 197) and the remainder used the term co‐production (*n* = 155). Due to the volume of definitions located, we excluded uncommon definitions by considering the average number of citations per year since the date of publication. We excluded 640 uncommon definitions that had fewer than one citation for every 5 years since publication. Included definitions (*n* = 475) were plotted on a timeline to establish when they were first published (Figure 3). While there has been an increasing trend in the number of definitions published during the last decade, we are observing a reversal of this trend in recent years (2016–2018).

### 3.3 Exploring a network of common definitions and relationships between definitions

**Figure 4:**
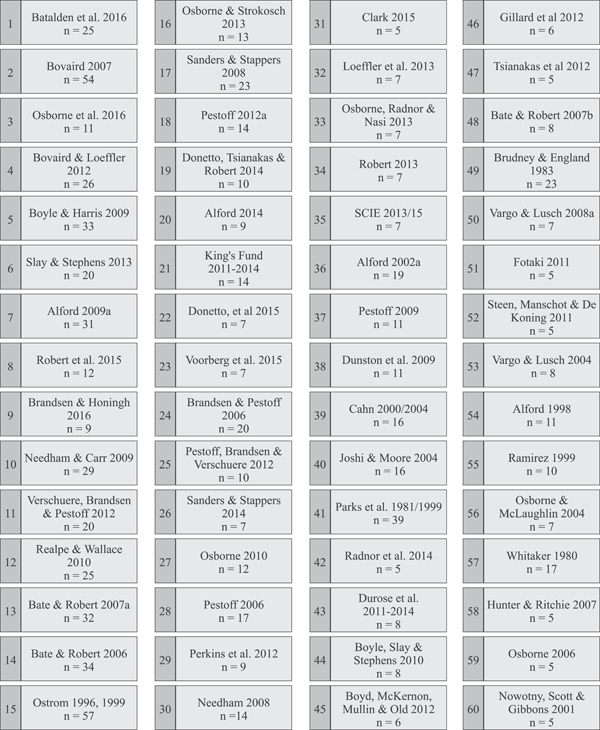
Most common definitions ranked by the average number of citations per year since publication. Definitions with at least five citations were ranked by considering the average number of citations per year since the date of publication

**Figure 5:**
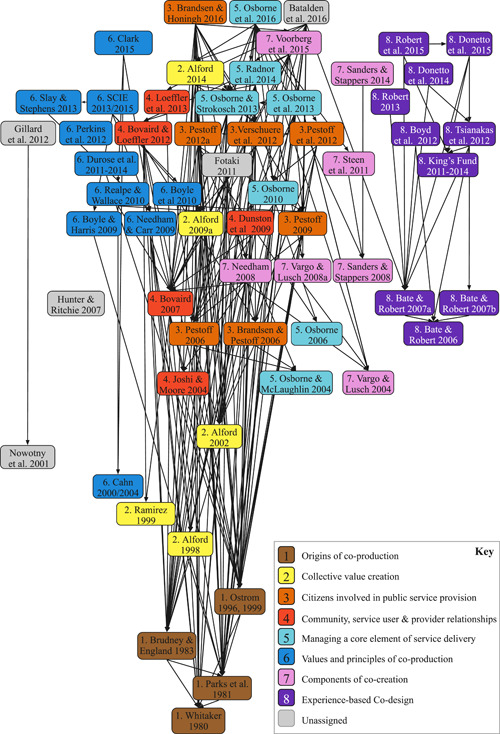
Network of co‐production and co‐design definitions as applied in health and social care. Definitions were added to Miro, a digital whiteboard, with each box representing a publication. A full description of how this figure was developed is provided within the main text. The positioning of the box on the y‐axis was decided by the year of publication, with the most recent presented at the top of the figure. Directional arrows represent a citation to another definition. These citation lines informed the positioning of each box on the *X*‐axis to group definitions together

The most common definitions (i.e., at least five citations) were then ranked by considering the average number of citations per year since the date of publication (Figure 4). Ranking these definitions accounts for publication date bias and provides an indication of which may be influential in future research in the context of health and social care. The top 60 ranked definitions were then visually mapped in order of publication date on a vertical axis, with each box in Figure 5 representing a cited definition. We read the original cited source to explore which earlier conceptual definitions were used to inform their theory of co‐production and/or co‐design and drew a line to represent each citation. These citation lines then determined the location of each definition on the horizontal axis on the network map. This resulted in a conceptual network map that presents a visual representation of how the most common definitions of co‐production and co‐design in the context of health and social care relate to—and have been informed by—each other (Figure 5). The labelling of each cluster was determined by reviewing the extracted definitions to identify key or common words that inform the core features for each cluster.

#### 3.3.1 Description of the network map

Within this network, there are eight prominent clusters of definitions that have been applied in health and social care. These are as follows: origins of co‐production; value creation; citizens involved in public service provision; community, service user and provider relationships; managing a core element of service delivery; values and principles of co‐production; components of cocreation; and experience‐based co‐design (EBCD).

1. **Origins of co‐production**
  Offering some of the first detailed definitions of co‐production, the articles within this cluster appear to serve as a foundation for the various concepts that have subsequently evolved. The most common definition in this cluster was Ostrom,^37^ who provided an initial, broad definition that established the core concept of how individuals outside of a service‐providing organization contribute to the delivery of that service. The label was assigned as the network lines demonstrate that these sources are typically cited as important works informing the development of the concept of co‐production. The core feature of these definitions is the emphasis on involvement or interaction of citizens and public agents^38^, ^39^ and that this can take place at an individual, group or collective level.^40^
2. **Collective value creation**
  Continuing with co‐production, the most common definition in this cluster was Alford,^41^ who builds upon the original definitions by specifying that co‐production is any active behaviour independent of but prompted by, a government agency that intentionally creates public or private value in the form of an output or outcome (p. 23). A further distinguishing feature is that this definition implies that initiating co‐production was the responsibility of service providers rather than service users. The label for this cluster was assigned as the core feature of the definitions in this cluster is an emphasis on the creation of value for a group or a collective.
3. **Citizens involved in public service provision**
  The most common definitions^3^, ^42^ in this co‐production cluster expand upon previously discussed definitions through the clarification of roles, with the service provider being distinguished as a ‘paid employee’ and co‐production requiring the ‘direct and active contribution’ of citizens.^3^ These definitions also place co‐production into a broader conceptual context, referring to the concept of co‐management and co‐governance.^42^ The core feature of these definitions is that they consider co‐production to be a mix of activities where both public service agents and citizens contribute to the production, provision and delivery of public services.
4. **Community, service user and provider relationships**
  Although primarily co‐production, a definition within this cluster^5^ identifies co‐design amongst a range of activities under what they term the ‘co‐production umbrella’ such as co‐commissioning, co‐planning, co‐prioritization, co‐financing, co‐design, co‐managing, co‐performing and co‐assessing. In the most common definition in this cluster, Bovaird^4^ defines ‘user and community co‐production’ as the provision of services through regular, long‐term relationships between professionalized service providers and service users or other members of the community (p. 847). The label of the cluster was chosen to reflect the core feature of these definitions, which emphasize the importance of relationships,^4^, ^43^, ^44^ dialogue^44^ and resource contributions.^4^, ^5^, ^43^
5. **Managing a core element of service delivery**
  Although primarily co‐production, the latest and most common definition in this co‐production cluster, Osborne et al.^45^ position co‐production within a broader range of activities such as the design, management, delivery and evaluation of public services. The label for this co‐production cluster was again based on the core feature for these definitions as highlighted by Radnor et al.,^46^ who consider co‐production to be a core element of service delivery; it is not an ‘add‐on’, but an unavoidable element that needs to be managed effectively. A distinguishing feature in this cluster is the presentation of three types of co‐production: consumer co‐production (improving the quality and impact of public services), participative co‐production (improving the planning of public services) and enhanced co‐production (bringing consumer experience together with participative planning to generate new approaches to public services).^47^
6. **Values and principles of co‐production**
  In the most common definition in this co‐production cluster, Boyle and Harris^48^ refer to an equal and reciprocal relationship between professionals, people using services, their families and their neighbours when delivering public services (p. 11). In contrast with other clusters, the core feature of these definitions is an acknowledgement that co‐production is difficult to define as it as an evolving concept^7^ and instead emphasizes values and principles of co‐production. More recently, Slay and Stephens^49^ and SCIE^7^ defined co‐production as a practice that aligns with six principles: assets‐based approach; building on people's existing capabilities; reciprocity and mutuality; engaging peer and personal networks alongside professionals to transfer knowledge; removing the distinction between professionals and recipients; and facilitating rather than delivering public service. In addition to these principles, this cluster of definitions raised the importance of considering principles of equality^48^, ^50^, ^51^ and power^52^, ^53^ and how this is balanced between people getting support and the professionals who deliver it^7^ at the various stages of the co‐production process.^54^
7. **A component of co‐creation**
  This cluster of definitions refers to both co‐production and co‐design. In the most common definition, Sanders and Stappers^12^ consider co‐design to be a specific instance of co‐creation (which they consider to be any act of collective creativity shared by two or more people) and define co‐design as collective creativity applied across the whole span of a design process between designers and people not trained in design (p. 6). The label of this cluster is informed by the core feature that these definitions consider co‐production and co‐design to either be a component of co‐creation,^55^, ^56^, ^57^ a specific instance of co‐creation^12^ or interchangeable with co‐creation.^10^ In the most recent definition, Voorberg et al.^10^ consider the term ‘co‐creation’ to refer to the involvement of citizens in the initiation and co‐design of public services, while the term ‘co‐production’ refers to the involvement of citizens in the implementation of public services.
8. **Experience‐based co‐design**

The final cluster was relevant to co‐design and labelled based on a six‐stage process referred to as EBCD in the definitions. In the most common definition in this cluster, Robert et al.^58^ state that applying this particular co‐design approach in the healthcare context represents a radical reconceptualization of the role of patients and a structured process for involving them throughout all stages of quality improvement (p. 3). A core feature of the definitions within this cluster is the emphasis on the importance of user experience^59^ and emotion^60^, ^61^, ^62^—as well as several concepts underpinning the change process (ownership and power)^63^—as being fundamental to the application of such approaches. A distinguishing feature is that this cluster refers to a form of participatory action research rather than a definition. The core feature of this cluster is the emphasis on subjective, personal feelings and experience of service users, carers and staff to understand and improve moments (‘touchpoints’) that form a person's overall experience of a service.^63^, ^64^

## 4 DISCUSSION

This review builds on previous work,^2^, ^10^, ^17^ and contributes by adopting a broad, inclusive approach using an extensive selection of databases to explore the concepts of co‐production and co‐design as they have been applied in health and social care. A range of definitions were identified ranging from generic outlines of the relationship between service providers and service users to elaborations on the qualities of these relationships and underpinning principles such as equity, power and trust. The findings of this review identify that co‐production and co‐design are evolving concepts, and this creates a level of uncertainty as to which definitions are relevant to which specific context.

Findings show that there has been an increase in articles exploring co‐production and co‐design in health and social care published in peer‐review journals during the last decade; this finding is consistent with recent reviews and research across multiple sectors.^1^, ^2^ However, our findings suggest that some of this increase in the health and social care sectors is being driven by articles using the terms co‐production or co‐design, but not involving (or reporting inclusion of) service users. Combined with the volume of definitions available and the high number of articles that do not provide a definition, this finding suggests that the core values underpinning co‐production and co‐design may be being diminished. Another potential interpretation is that the reporting of co‐production and co‐design in the peer‐review literature needs to be improved. Both interpretations support the concern raised by SCIE^7^ and Williams et al.,^9^ in relation to the misappropriation of the term ‘co‐production’. There is a valid argument that time spent debating the definitions of concepts such as co‐production or co‐design could be better spent listening to service users. However, we recommend that both should take place with service users, because if concepts that set out to share power are (inadvertently) misused, not only may assets and resources be wasted through the implementation of ineffective or inefficient service solutions but mistrust in health and social care services and/or dilution of the impact of genuine co‐production and co‐design approaches may also result.

Another important finding is the identification of the discrete clusters of sources and definitions that underpin the application of co‐production and co‐design in the health and social care sectors. This analysis suggests that, in this context at least, co‐design is not a subsidiary of co‐production, but a separate and distinct concept. While the terms may sometimes have been considered interchangeable,^10^ the development of co‐design in health and social care appears to have been largely separate to that of co‐production. However, the network map shows signs of these concepts entwining in recent publications. Two articles worth noting are those of Batalden et al.^65^ and Osborne et al.,^45^ both of which connect the co‐production and co‐design clusters. These findings suggest that considering co‐production as an umbrella term may be too constraining. However, others have argued that defining the various ‘co’‐terms within strict boundaries may no longer be feasible and may serve to cause confusion and create barriers to engagement with those we seek to work with.^32^ This review found that there are a number of value‐based frameworks and guides available, particularly for co‐production.^7^, ^66^, ^67^, ^68^ Despite this, this review highlights how values and principles are infrequently discussed and seemingly rarely applied. However, we are beginning to witness their discussion in peer‐review discussion papers in co‐production^69^ and co‐design.^70^ This ongoing shift from defining concepts and distinguishing contexts towards agreeing values and principles that are considered essential is a promising way forward. More clearly articulating the shared values and principles underpinning both co‐production and co‐design is recommended.

## 5 LIMITATIONS

Even with the broad scope adopted, it is likely that our review will have missed relevant articles that do not mention ‘health’, ‘social’, ‘public service’ or ‘public sector’ in their title, abstract or key words.^71^ The inclusion of only English‐language articles will have resulted in an under‐representation of co‐production and co‐design studies in non‐English‐speaking countries. Furthermore, this review set out to explore peer‐review research and so this is only the ‘tip of the iceberg’ in terms of co‐production and co‐design. It is likely that many co‐production or co‐design projects will be published in forms other than peer‐reviewed articles within English‐language journals. Further work should explore how concepts related to co‐production and co‐design are described and conceptualized in other countries. Finally, as we have focused on interconnections between common definitions within health and social care and that have involved service users, we may not have captured all conceptual linkages. For example, as co‐creation was not a focus of this review and as the term was not included in the search strategy, it is likely that we have missed potentially relevant articles for this cluster, which warrants further exploration by building upon our strategy and that of similar reviews.^10^, ^15^

## 6 CONCLUSIONS

This review has synthesized definitions that have been cited in research to help conceptualize co‐production and co‐design. The main contribution of this review is that it provides an overview and a map of the definitions of co‐production and co‐design that have been most commonly applied within the health and social care sectors. This scoping review is the first to systematically gather and map the interconnections between these definitions in this context. For those new to co‐production and co‐design (as well as those who are experienced), this mapping can help improve the identification of relevant definitions and aide the translation of their core elements into practice. This review provides a knowledge resource to help explore and understand the different and connecting concepts within this broad and growing field.

With so many potential definitions, including those widely cited and established, co‐production and co‐design remain evolving concepts as applied in the context of health and social care. It is recommended that the focus shift towards advocating for and operationalizing the underlying and aligning values and principles of co‐production and co‐design that need to be translated into practice.

## CONFLICTS OF INTEREST

The authors declare no conflicts of interest.

## AUTHOR CONTRIBUTIONS

All authors including Daniel Masterson, Kristina Areskoug Josefsson, Glenn Robert, Elisabeth Nylander and Sofia Kjellström made substantial contributions to the conception and design of the study, the analysis and interpretation of the data, drafting and revision of the article and final approval of the version to be submitted. Daniel Masterson takes responsibility for the integrity of the work as a whole.

## Supporting information

Supporting information.

Supporting information.

Supporting information.

Supporting information.

## Data Availability

The full search strategy and synthesis procedure has been provided to ensure replicability. The data that support the main findings of this study are available in the full text of this article. The database containing the included articles will be made available in Figshare at 10.6084/m9.figshare.19248620 following an embargo from the date of publication to allow for the next phase of the study to be completed.
