# Supplementary material for: Mapping definitions of co‐production and co‐design in health and social care: A systematic scoping review providing lessons for the future
Source: Health Expect. 2022 Mar 23;25(3):902–13. doi: 10.1111/hex.13470 (PMC9122425; doi:10.1111/hex.13470)
Supplement: Supplementary file 2 — Supporting information. [file HEX-25--s004.docx]

# Supplementary file 2. Key words from previous reviews and studies recommended by experts in relevant fields

| **Seminal articles informed by experts** | **Key words** |
| --- | --- |
| Alford (2014) | Co-production |
| Batalden et al. (2016) | Co-production |
| Batalden (2018) | Co-production |
| Bovaird (2007) | Co-production, Engagement, participation |
| Durose et al (2017) | Co-production, appreciative inquiry |
| Farr (2017) | co-design, collaboration, collective reflexivity, co-production, participation, power, user involvement |
| Osborne et al. (2016) | Co-production, public services reform, active citizens, active communities, public service-dominant logic, co-creation, public value |
| Robert et al. (2015). | Co-design |
| **Previous reviews and research** |  |
| Allen, Needham, Hall, and Tanner (2018) | Co‐production, co‐research, social care |
| Almeida, Cappelli, Maciel, and Mahecha (2018) | Co-production |
| Bench, Eassom, and Poursanidou (2018) | Consumer, critical care, health research, improvement science, patient and public involvement |
| Bunn et al. (2018) | Shared decision making, Person-centred care |
| Crompton (2018) | Co‐production, decision‐making, public engagement, public participation |
| Clarke, Waring, and Timmons (2018) | Coproduction of knowledge, inclusivity, interaction rituals, situated practice, translational research |
| De Weger et al. (2018) | Community engagement, Citizen engagement, Community participation |
| Flinders and Wood (2018) | Co‐production, ethnography, governance, narratives, resistance |
| Gale, Brown, and Sidhu (2018) | Co‐production, decentred theory, public health workforce |
| Gillespie, Magee, White, and Stewart (2019 | Qualitative Co-production |
| Hampshaw, Cooke, and Mott (2018) | Delphi study, Knowledge transfer, Dissemination, Knowledge translation, Research derived actionable tool (RDAT) |
| Hoekstra et al. (2018) | Collaborative research partnerships, Integrated knowledge translation, Community-based participatory research, Stakeholder engagement |
| Honingh et al. (2018) | Co-production, parental involvement, participation |
| Hughes and Duffy (2018) | Co-production, participatory research, PPI, public and patient involvement, social work, user-controlled research, user-led research |
| Jaspers and Steen (2018) | Co-production; public values; social care; value conflicts |
| Jennings, Slade, Bates, Munday, and Toney (2018) | Patient and public involvement (PPI), Mental health research, Collaborative data analysis, Co-production |
| Jo and Nabatchi (2018) | Coproduction; effects of coproduction; empowerment; issue awareness; trust in professionals |
| Jull, Giles, and Graham (2017) | Community-based participatory research, Integrated knowledge translation, Engagement, Collaboration, Health systems, Co-creation |
| Kavcic, Pahor, and Domajnko (2015) | User involvement, Healthcare, Co-production, Choice, Voice |
| Kislov et al. (2018) | CLAHRC, Evaluation, Collaboration, Learning health systems, Co-production, Knowledge mobilisation, Implementation |
| La Place and Corlyon (2014) | Research, Inclusion, Service use |
| Laycock et al. (2018) | Quality of care, continuous quality improvement, primary health care, participatory research, integrated knowledge translation |
| McMullin (2018) | Coconstruction, coproduction |
| Pilgrim (2018) | Mental health services, Co-production |
| Richardson, Durose, and Perry (2019) | Citizen participation, hybridity, transdisciplinarity |
| Rütten et al. (2017) | Structure and agency, population health intervention research, knowledge exchange, co-production |
| Waring et al. (2018) | Organisational politics, Political skill, Political astuteness, Leadership, System change, Qualitative, Ethnography |
| Weaver (2018) | Co‐production, criminal justice, decentered theory, governance, user voice |
| Voorberg et al. (2015) | Co-creation, co-production, public-sector, innovation, social innovation |
