# Supplementary material for: Mapping definitions of co‐production and co‐design in health and social care: A systematic scoping review providing lessons for the future
Source: Health Expect. 2022 Mar 23;25(3):902–13. doi: 10.1111/hex.13470 (PMC9122425; doi:10.1111/hex.13470)
Supplement: Supplementary file 3 — Supporting information. [file HEX-25--s001.docx]

### **Supplementary file 3:** Search strategy piloting key terms

| **#** | **Search terms: Concept [All fields]** | **PubMed** | **Scopus** |
| --- | --- | --- | --- |
| 1 | Co-produc* OR coproduc* OR co-design* OR codesign* OR co-creat* OR cocreat* OR Codeliver* OR Co-deliver* OR Service User OR co-evaluat* OR coevaluat* | 15 316 | 910 666 |
| 2 | Co-produc* OR coproduc* OR co-design* OR codesign* OR co-creat* OR cocreat* OR Codeliver* OR Co-deliver* OR co-evaluat* OR coevaluat* | 7750 | 155 502 |
| 3 | Co-produc* OR coproduc* OR co-design* OR codesign* OR co-creat* OR cocreat* OR Codeliver* OR Co-deliver* | 7677 | 154 652 |
| 4 | Co-produc* OR coproduc* | 3884 | 68 160 |
| 5 | co-design* OR codesign* | 611 | 43 971 |
| 6 | AND Health OR Social |  |  |
| 7 | 4 OR 5 | 2054 | 23 183 |
| 8 | **(AND NOT)** Proton OR chemistry OR physics OR biology OR biochemical OR engineer* OR biomass OR chemic* OR adsorption OR bioproduct* OR osmosis OR microalgae OR genetic OR genomic OR enzyme OR ecosystems OR hydrogen | - | - |
| 9 | 4 AND NOT 8 | 2687 | 16 792 |
| 10 | 5 AND NOT 8 | 491 | 12 215 |
| 11 | Co-produc* OR coproduc* OR co-design* OR codesign* | 4457 | 111 366 |
| 12 | 1 AND NOT 8 | 8815 | 516 933 |
| 13 | 11 AND NOT 8 | 1655 | 28 615 |
| 14 | 11 with YEAR LIMIT to 2017 | 672 | 18 502 |
| 15 | United Kingdom OR Britain OR England OR Sweden |  |  |
| 16 | **(AND NOT)** Proton OR chemistry OR physics OR biology OR biochemical OR engineer* OR biomass OR chemic* OR adsorption OR bioproduct* OR osmosis OR microalgae OR genetic OR genomic OR enzyme OR ecosystems OR hydrogen OR industry OR Brand |  |  |
| 17 | Replicate: Coproduction OR co-production OR coproduce OR co-produce OR coproducing OR co-producing | 1949 | 9385 |
| 18 | Replicate: Cocreation OR co-creation OR cocreate OR co-create OR cocreating OR cocreating | 454 | 6590 |
| 19 | Replicate: Codesign OR co-design OR codesigning OR co-designing | 483 | 10398 |
| 20 | Coproduction OR co-production OR coproduce OR co-produce OR coproducing OR co-producing OR Cocreation OR co-creation OR cocreate OR co-create OR cocreating OR cocreating OR Codesign OR co-design OR codesigning OR co-designing | 2817 | 100 636 |
| 21 | 20 AND NOT 16 | 1412 | 23 157 |
| 22 | 1 AND NOT 16 | 8579 | 333 921 |
| 23 | **AND NOT** chemistry OR physics OR biology OR biochemical OR engineer* OR biomass OR chemic* OR bioproduct* OR osmosis OR microalgae OR genetic OR genomic OR enzyme OR ecosystems OR hydrogen OR industry OR Brand OR Algebra OR math* OR economics OR software OR gene* |  |  |
| 24 | 11 AND NOT 26 | 1398 | 7185 |
| 25 | **AND NOT** chemistry OR physics OR biology OR biochemical OR engineer* OR biomass OR chemic* OR bioproduct* OR osmosis OR microalgae OR genetic OR genomic OR enzyme OR ecosystems OR hydrogen OR industry OR Brand OR Algebra OR math* OR economics OR software OR gene |  |  |
| 26 | 11 AND NOT 25 | 1388 | 11 290 |
| 27 | 11 AND 15 AND NOT 23 | 298 | 1939 |
| 28 | 11 AND 6 AND NOT 23 | 869 | 4 418 |
| 29 | 11 AND 6 AND 15 AND NOT 23 | 272 | 1640 |
| 30 | **AND** Health OR Social OR “public services” OR “public service” |  |  |
