# Supplementary material for: Mapping definitions of co‐production and co‐design in health and social care: A systematic scoping review providing lessons for the future
Source: Health Expect. 2022 Mar 23;25(3):902–13. doi: 10.1111/hex.13470 (PMC9122425; doi:10.1111/hex.13470)
Supplement: Supplementary file 4 — Supporting information. [file HEX-25--s002.docx]

**Supplementary file 4.** Search strategy for each database

| **Search terms to be included in title, abstract or key words** |
| --- |
| (Co-produc* OR coproduc* OR co-design* OR codesign*)  **AND** (health OR social OR "Public Service*" OR “public sector”)  **AND NOT** (engineer* OR biomass OR bioproduct* OR osmosis OR microalgae OR ecosystems OR hydrogen OR Brand OR Algebra) |

Note: At the time of our search (March 2019) and writing, the searched databases lacked controlled vocabulary or subject headings for ‘co-production’, so the decision was made to only search in the title, abstract, and key word (subjects) fields.

Excluding irrelevant contexts was aided through the search strategy (engineer* OR biomass OR bioproduct* OR osmosis OR microalgae OR ecosystems OR hydrogen OR Brand OR Algebra). An alternative to incorporating these terms could have been to only search subject specific databases (e.g. MEDLINE) but this may have omitted a significant number of relevant papers.

**CINAHL with Full Text (EBSCOHost) – 588 hits on March 18, 2019**

TI ( ( co-produc* OR coproduc* OR co-design* OR codesign* ) AND ( health OR social OR “public service*” OR “public sector*” ) NOT ( engineer* OR biomass OR chemic* OR bioproduct* OR osmosis OR microalgae OR ecosystems OR hydrogen OR brand OR algebra ) ) OR AB ( ( co-produc* OR coproduc* OR co-design* OR codesign* ) AND ( health OR social OR “public service*” OR “public sector*” ) NOT ( engineer* OR biomass OR chemic* OR bioproduct* OR osmosis OR microalgae OR ecosystems OR hydrogen OR brand OR algebra ) )

**Limiters** - Peer Reviewed; English Language

**Cochrane Central Register of Controlled Trials (Wiley) – 47 hits on March 19, 2019**

(co-produc* OR coproduc* OR co-design* OR codesign*):ti,ab,kw AND (health OR social OR “public service*” OR “public sector*”):ti,ab,kw NOT (engineer* OR biomass OR chemic* OR bioproduct* OR osmosis OR microalgae OR ecosystems OR hydrogen OR brand OR algebra):ti,ab,kw

**MEDLINE (EBSCOHost) – 793 hits on March 18, 2019**

TI ( ( co-produc* OR coproduc* OR co-design* OR codesign* ) AND ( health OR social OR “public service*” OR “public sector*” ) NOT ( engineer* OR biomass OR chemic* OR bioproduct* OR osmosis OR microalgae OR ecosystems OR hydrogen OR brand OR algebra ) ) OR AB ( ( co-produc* OR coproduc* OR co-design* OR codesign* ) AND ( health OR social OR “public service*” OR “public sector*” ) NOT ( engineer* OR biomass OR chemic* OR bioproduct* OR osmosis OR microalgae OR ecosystems OR hydrogen OR brand OR algebra ) )

**Limiters** – English Language

**PsycINFO (ProQuest) – 403 hits on March 18, 2019**

ti((co-produc* OR coproduc* OR co-design* OR codesign*) AND (health OR social OR "public service*" OR "public sector*") NOT (engineer* OR biomass OR chemic* OR bioproduct* OR osmosis OR microalgae OR ecosystems OR hydrogen OR brand OR algebra)) OR ab((co-produc* OR coproduc* OR co-design* OR codesign*) AND (health OR social OR "public service*" OR "public sector*") NOT (engineer* OR biomass OR chemic* OR bioproduct* OR osmosis OR microalgae OR ecosystems OR hydrogen OR brand OR algebra))

**Limits** – Peer reviewed; Language: English

**PubMed (legacy) – 898 hits on March 18, 2019**

Search (((co-produc*[Title/Abstract] OR coproduc*[Title/Abstract] OR co-design*[Title/Abstract] OR codesign*[Title/Abstract])) AND (health[Title/Abstract] OR social[Title/Abstract] OR “public service*”[Title/Abstract] OR “public sector*”[Title/Abstract])) NOT (engineer*[Title/Abstract] OR biomass[Title/Abstract] OR chemic*[Title/Abstract] OR bioproduct*[Title/Abstract] OR osmosis[Title/Abstract] OR microalgae[Title/Abstract] OR ecosystems[Title/Abstract] OR hydrogen[Title/Abstract] OR brand[Title/Abstract] OR algebra[Title/Abstract])

**Filters:** English

**Scopus (Elsevier) – 2462 hits on March 18, 2019**

( TITLE-ABS ( co-produc* OR coproduc* OR co-design* OR codesign* ) AND TITLE-ABS ( health OR social OR "public service*" OR "public sector*" ) AND NOT TITLE-ABS ( engineer* OR biomass OR chemic* OR bioproduct* OR osmosis OR microalgae OR ecosystems OR hydrogen OR brand OR algebra ) ) AND ( LIMIT-TO ( LANGUAGE , "English" ) )
